# The effect of case-based mobile virtual patient application on students’ academic achievement in clinical reasoning skills

**DOI:** 10.1080/10872981.2024.2322223

**Published:** 2024-03-06

**Authors:** Levent Çetinkaya, İ̇lke Keser, Serkan Yildirim, Hafize Keser

**Affiliations:** a Department of Computer Education and Instructional Technology, Canakkale Onsekiz Universty, Canakkale, Türkiye; b Department of Physiotherapy and Rehabilitation, Faculty of Health Sciences, Gazi University, Ankara, Türkiye; c Department of Computer Education and Instructional Technology, Ankara University, Ankara, Türkiye; d Faculty of Educational Sciences, Department of Computer Education and Instructional Technology, Ankara University, Ankara, Türkiye

**Keywords:** Clinical reasoning, clinical skills, practical application, virtual patient, mobile learning

## Abstract

This mixed-method study aims to determine the effect of the use of mobile virtual patient application with narrated case-based virtual patients as an assistive technology on students’ clinical reasoning skills. It makes a notable contribution by exploring the impact of mobile virtual patient applications on healthcare students’ clinical skills and their preparation for real-world patient care. In addition, the accuracy of the analysis results regarding the effect on student achievement was analyzed with a second dataset tool, and thus, aiming to increase reliability by verifying the same research question with a different quantitative analysis technique. In the qualitative part of the study, students’ views on the implementation were collected through an open-ended questionnaire and the data were subjected to content analysis. An achievement test was also developed to determine the development of students’ clinical reasoning skills, which revealed that each of the learning environments had different outcomes regarding students’ achievement and that supporting the traditional environment with the mobile virtual patient application yielded better results for increasing students’ achievement. Students’ opinions about the mobile virtual patient application and the process also support the increase in academic achievement aimed at measuring clinical reasoning skills. The content analysis showed that the students, who generally reported multiple positive factors related to the application, thought that the stories and cases presented created a perception of reality, and they especially highlighted the contribution of the application to learning the story organization. Based on all these results, it can be said that the application supports clinical reasoning, provides practical experience, improves academic achievement, and contributes positively to motivation.

## Introduction

Health field education includes both theoretical knowledge and practical skills and generally includes an intensive curriculum. The acquisition of practical skills within the intensive educational process is very important for students to be able to plan and manage their work processes after graduation. To accomplish this objective, students must cultivate the ability to diagnose and manage clinical issues, a process often referred to as the development of clinical reasoning skills [1]. This development is particularly crucial given the complex nature of clinical environments where theoretical knowledge must be applied effectively [2]. The acquisition of these skills is crucial for students to acquire not only clinical skills but also the essential elements of their professional selves [3]. Clinical reasoning, which is seen as a core competency that all health professionals should develop [4], generally refers to the thought processes and steps involved in making a clinical judgment [5,6]. Effective clinical reasoning involves integrating both theoretical knowledge and practical experience [7]. Clinical reasoning, also defined as a decision-making process guiding actions [8,9], is a context-dependent skill necessary for best professional performance [6,10,11]. Therefore, it’s crucial to embed clinical reasoning skills early in medical education, using innovative and active learning methods [12]. Experts often use clinical reasoning through clinical pattern recognition, through which earlier clinical presentations are related to the patient’s current condition [13–15]. In contrast to professionals, less-experienced professionals make decisions based almost entirely on theoretical knowledge and gain clinical experience over time [16–18]. Students should have a very good grasp of the theoretical knowledge related to the health field they are studying and be prepared to use it effectively during clinical problem solving. Active learning methods, such as the use of virtual patients, have been shown to enhance the development of these crucial skills [19]. Experts agree that clinical reasoning skills should be included in the early years of profession and refined through clinical practice in later years [20]. However, many students may have limited contact and experience with patients before graduation. Therefore, it should be ensured that they gain clinical reasoning skills in the early stages of their education and see their possible mistakes in the process. It should also be ensured that the experienced errors are minimized and the students trained in the field of health should use problem solving and metacognitive strategies during their education, which are the necessary skills for their autonomous individual careers after graduation [21]. Otherwise, they run the risk that health professionals who graduate with poor clinical reasoning skills will not be able to provide effective and safe health services to patients. To reduce this risk, active learning methods need to be incorporated into curricula so that students can acquire clinical reasoning skills and achieve the targeted learning objectives [12].

Active learning methodologies can support the diversification of teaching and learning techniques in line with the needs. Especially when faced with the challenges in health sciences education, the use of technology-assisted learning is seen as important to optimize instructional processes [22]. Such technologies, including mobile applications and virtual environments, are increasingly being recognized for their effectiveness in enhancing learning outcomes in medical education [23]. Technology-assisted learning is generally welcomed by students and has the potential to facilitate active learning [24]. In particular, virtual patient applications are viewed as a technological methodology and an important alternative for developing clinical reasoning competencies [5]. Studies have shown that virtual patient simulations can significantly improve clinical skills, indicating their utility in medical training [19]. In general, studies show that virtual patient tools can effectively complement existing teaching and can be a useful tool, especially when face-to-face learning opportunities are limited [25]. Increasingly popular in healthcare education, virtual patient teaching tools allow students to realistically engage in clinical reasoning with patients in a safe environment [5,18,26–28]. This engagement is crucial as it offers a practical approach to understanding complex medical scenarios [29]. Although these tools are not viewed as a sufficient strategy for teaching clinical reasoning alone and cannot replace real clinical practice, they are very important in terms of skill acquisition [18,19,30]. Furthermore, the incorporation of virtual patients in educational settings has been associated with enhanced student motivation and better preparation for real-world clinical challenges [7].

In healthcare education, presenting real patients or realistic patient scenarios to students is extremely important for their development in terms of learning new medical procedures by doing, acquiring new skills, and treatment planning. This practical exposure is crucial for bridging the gap between theoretical knowledge and clinical application, a key aspect of medical education [23]. However, due to the problems encountered in the implementation of practical courses in many universities, students have difficulties in reflecting the theoretical knowledge they have acquired in the course to their performance in practical exams and preparing in advance for the situations they will encounter in the clinical environment. Virtual patient applications have emerged as a solution to these challenges, providing interactive and realistic scenarios for students to practice and hone their clinical reasoning skills [19]. Virtual patient applications, which have gained critical importance in overcoming these difficulties, should be associated with real cases and presented with narrated scenarios to give a sense of reality. This approach enhances the learning experience by providing a more immersive and engaging environment for students [29]. When students start working with virtual patients blended with stories, it contributes positively to their better preparation for later curriculum experiences and their potential to help real patients [31]. On the other hand, presenting a context-dependent skill with narrated real cases without space and time limitations can contribute to supporting traditional teaching processes. Mobile applications, in this regard, offer flexibility and accessibility, making them a valuable addition to healthcare education [32,33]. Accordingly, mobile applications can be used as a supportive or alternative teaching environment to solve the limitations in traditional learning processes. Since mobile learning supports different learning environments, it improves the learner’s research ability by creating context awareness [32]. In today’s world of digitization, mobile applications must be aligned with the essence of mobile learning, that is, improve connectivity and flexibility between people, contexts, content, times, places, and learning methods [33,34]. Although interest in mobile learning and research has increased worldwide [35], one of the biggest and most important problems is that mobile learning solutions have not been widely integrated into educational practices. While the increasing adoption of such technologies in medical education indicates a positive trend towards more interactive and effective learning experiences [36], there remains a need for studies to help equip future healthcare professionals with high-level knowledge and skills in learning and practicing new medical procedures and planning treatment. This study, which was carried out to address this need, aimed to determine the effect of the use of a mobile virtual patient application with narrated case-based virtual patients as an assistive technology in the education and training process on students’ clinical reasoning skills and to find out students’ views on the application process. To this end, we sought the answers to the research questions below:

1. Does the use of the mobile virtual patient application as an assistive technology in a traditional environment significantly impact students’ academic achievement scores for clinical reasoning skills?
2. What are the students’ opinions on the mobile virtual patient application and the study process?

## Method

### Research design

Using a mixed-method approach, this study leveraged the strengths and mitigated the limitations of both qualitative and quantitative research methodologies, aiming for a holistic understanding by integrating results from both methods [37,38]. Specifically, we adopted an explanatory mixed-method design, wherein qualitative data were employed to corroborate or refine quantitative findings [37]. Within this framework, a quasi-experimental pretest-posttest control group design was used to evaluate the efficacy of the mobile virtual patient application when combined with the traditional learning environment. This quasi-experimental approach, known for effectively delineating cause-and-effect relationships between variables, involves administering the pre- and post-tests to determine the impact on the independent variable [39]. Details of this design, where experimental and control groups are delineated based on preliminary measurements and criteria, are provided in Table 1.

**Table 1.** Quasi experimental design research outline.

Group	Pre experimental	Experimental process	Post experimental
Experimental	Pretest (AT-17)	Traditional Environment and Mobile Virtual Patient App.	Posttest (AT-32)
Control		Traditional Environment	

*Note. AT=Achievement Test; Independent variable: Traditional environment complemented with mobile virtual patient application as supportive technology, and a standalone traditional environment; Dependent variable: Achievement.

In this stage, which constitutes the quantitative part of the research, the effect of case analysis on academic achievement of the students through the mobile virtual patient application in addition to the traditional curriculum was compared. The change in students’ academic achievement was ascertained by means of an achievement test consisting of 32 questions developed by the researchers. The students who were to constitute the experimental and control groups were not included in the achievement test development process, and 17 questions were asked in the pretest application, taking into account the assumption that the prior knowledge levels of the students who were going be confronted with these questions for the first time would be low and considering possible limitations. In the posttest stage of the study, the entire test, which included the questions asked in the pretest stage and included 15 questions that the students would encounter for the first time, was administered. The main purpose here was to reduce the limitation caused by the risk of students’ remembering the questions asked in the pretest at the posttest stage and to increase the reliability of the study. In addition, it was aimed to verify the results obtained through quantitative analysis with a second dataset. The other dimension of the study was designed as a case study, following the qualitative research tradition. With the case study, existing examples were revealed by asking the question of ‘how’ [40], and student opinions about the process were obtained in this part of the study.

### Study group

Students in the field of Physiotherapy and Rehabilitation, who traditionally engage in an active learning process at the patient bedside in the ward or outpatient clinic, were determined as the participants of the study. Criterion sampling, a purposeful sampling method, was used to select the study group. The following criteria were taken into account in the selection: ensuring the continuity of the experimental procedures, ease of access to the subjects, close levels of prior knowledge about the course, the availability of the technological infrastructure required for the application, and voluntariness. Among the students who attended the Neurological Rehabilitation course taught by the same faculty member, those who met the criteria were evaluated as the experimental group, while those who did not meet the criteria were considered as the control group.

As can be seen in Table 2 above, conducted with 52 students (13 female and 13 male students in the experimental group and 14 female and 12 male students in the control group), the study was terminated with the participation of all students in the posttest intervention.

**Table 2.** Gender distribution of the study group.

Study groups	Gender				Total	
	Female		Male			
	*f*	%	*f*	%	*f*	%
Control Group	14	53.8	12	46.2	26	50.0
Experimental Group	13	50.0	13	50.0	26	50.0
Total	27	51.9	25	48.1	52	100.0

### Intervention

In the study conducted with the permission of Gazi University Ethics Commission numbered E.136652, the face-to-face instruction for all the participants were delivered by the same lecturer by following the common curriculum. Additionally, the experimental group was instructed to use the mobile virtual patient application as a supplemental tool to the traditional curriculum. The lecturer completed the lecturing and assessment processes without knowing which group the students were in. For the experimental group, specific instructions were provided on how to navigate and interact with the mobile application. These instructions included guidelines on accessing and interpreting clinical scenarios, responding to embedded questions, and utilizing feedback mechanisms within the app.

The students in the experimental group were given the flexibility to use the mobile application as per their convenience. It was observed that the average usage time for the application was approximately 4 hours and 13 minutes [min 1 h 23 m, max 11 h 13 m]. This variation in usage time reflects the self-directed and diverse nature of the learning experience, indicating that students engaged with the application according to their individual learning needs and schedules. The study groups were not intervened in any way during the process and the data obtained were analyzed after the end of the course. Throughout the intervention, the frequency and duration of each student’s interaction with the application were monitored to assess engagement and compliance with the study protocols.

#### Creation and implementation of the achievement test

The study was conducted within the scope of the Neurological Rehabilitation course in the 3^rd^-year curriculum of the physiotherapy and rehabilitation program, which has intensive learning outcomes for the students’ clinical reasoning skill development. In line with the outcomes of the Neurological Rehabilitation applied course, with at least 3 questions prepared by the field expert to measure each outcome, a total of 35 questions were prepared. Before the pre-test piloting, the clarity, comprehensibility and appropriateness of the questions to the course outcomes were evaluated by three faculty members teaching the same course at three different universities as well as an academic who is an expert in the field of assessment and evaluation. The multiple-choice questions, which were prepared to reflect the learning outcomes in the curriculum, were arranged in such a way that they were clear and had a single correct answer for the final decision regarding the medical case. After the content validity of the questions was ensured after the evaluations of the experts, the item analysis was carried out with the participation of 121 students continuing their education in two different universities at the same education level as well as the students of the university where the study would be conducted. For each question, five options were presented to the students and 1 point was given for each correct question, and 0 point was given for each incorrect or unanswered question. Kuder Richardson-20 (KR-20) reliability was applied to examine the internal consistency between the test scores obtained and the relationship between the scores obtained from the test items, and the total score of the test was calculated by item total score correlation. Items with an item-total correlation of .30 and higher are generally thought to discriminate individuals well, and a test reliability coefficient of .70 and higher is generally sufficient for the test score reliability [39,41]. In light of the analyses, 3 items with an item-total correlation of .30 and below were removed and a 32-item achievement test was created, starting with the questions with the highest item-total correlation. The reliability value of the achievement test, comprising questions with item total correlations between .30–.90 and a Mean Discrimination Index of .41, was calculated as .83.

The common questions asked in the pretest and posttest stages of the study were determined by the field expert and reliability analyses were also conducted on these questions. The calculation performed on the achievement test form consisting of a total of 17 questions with at least 2 and at most 3 questions to measure each outcome revealed that the item-total correlation was in the range of .32–.83. The reliability value of the achievement test, which consisted of questions with a Mean Discrimination Index of .52, was calculated as .78 in KR-20. In the posttest stage of the study, an achievement test with 32 items, which included the questions in the achievement test given in the pretest stage and whose reliability was tested before, was administered.

In addition to the structured achievement test, students’ engagement with the mobile virtual patient application was closely monitored. This included tracking their interactions with the application and the cases they worked on. Real-time feedback was provided to students during their case studies through the application, offering guidance and support in their clinical reasoning process. This feedback mechanism within the application played a crucial role in enhancing students’ learning experience, as it allowed them to receive immediate and relevant feedback on their decisions and actions.

#### Creation and inclusion of mobile virtual patient application content

In the study, a mobile teaching system that includes a content management system module where academic staff can develop mobile learning content to be used in their own courses and mobile learning system modules that will support the development of students’ clinical decision-making and treatment planning skills was used. This system facilitated an interactive learning environment where students could actively engage with the virtual patient cases. By interacting with these cases, students were able to apply their theoretical knowledge to practical scenarios, enhancing their understanding of complex medical conditions. The mobile virtual patient application, developed by Yildirim [42] with a design-based research strategy within the scope of his doctoral dissertation, was designed as a content management system specific to the health field where faculty members can upload and manage their own content. The application’s interactive case content allowed students to simulate real-world clinical decision-making processes, providing a dynamic and engaging learning experience. This hands-on approach was instrumental in developing students’ clinical skills and confidence.

Throughout their interaction with the mobile virtual patient application, students received instant feedback on their decisions, enabling them to learn from their actions and refine their clinical reasoning. This feedback was crucial in providing a supportive learning environment, where students could experiment and learn without the fear of real-world consequences. The engagement with the application was further enhanced by its user-friendly design, making it accessible and appealing to students. This ease of use encouraged frequent and sustained use of the application, ensuring that students had ample opportunity to practice and hone their skills.

Overall, the inclusion of the mobile virtual patient application in the curriculum represented a significant advancement in medical education, offering a novel and effective way for students to develop critical clinical skills. The application’s integration into the course not only supported traditional learning methods but also provided a unique platform for students to experience and learn in a way that was both innovative and relevant to their future professional practice.

This system, where interactive case content can be created and student interaction can be evaluated, was developed to support the development of students’ clinical decision-making and treatment planning skills. In addition to all these features, the use of the mobile teaching system, which allows the creation of interactive cases with its health-specific structure and is designed in a way that students can easily use, was deemed appropriate based on expert opinions. First of all, 10 case scenarios with the same neurological disease diagnosis but with different clinical features that can be encountered in the clinic were created to improve students’ clinical decision-making competencies, taking into account the curriculum and achievements. In the process of creating these scenarios, the opinions of 1 expert in the field of Physiotherapy and Rehabilitation, 3 experts in the field of Physiotherapy and Rehabilitation in the evaluation of the suitability of the scenarios, as well as 3 experts in the field of Computer Education and Instructional Technology in the development and transfer of scenarios to the environment were elicited. The design to be presented to the students was finalized by applying expert opinions about the created scenarios and their compatibility with the mobile learning system.

**Figure 1.**
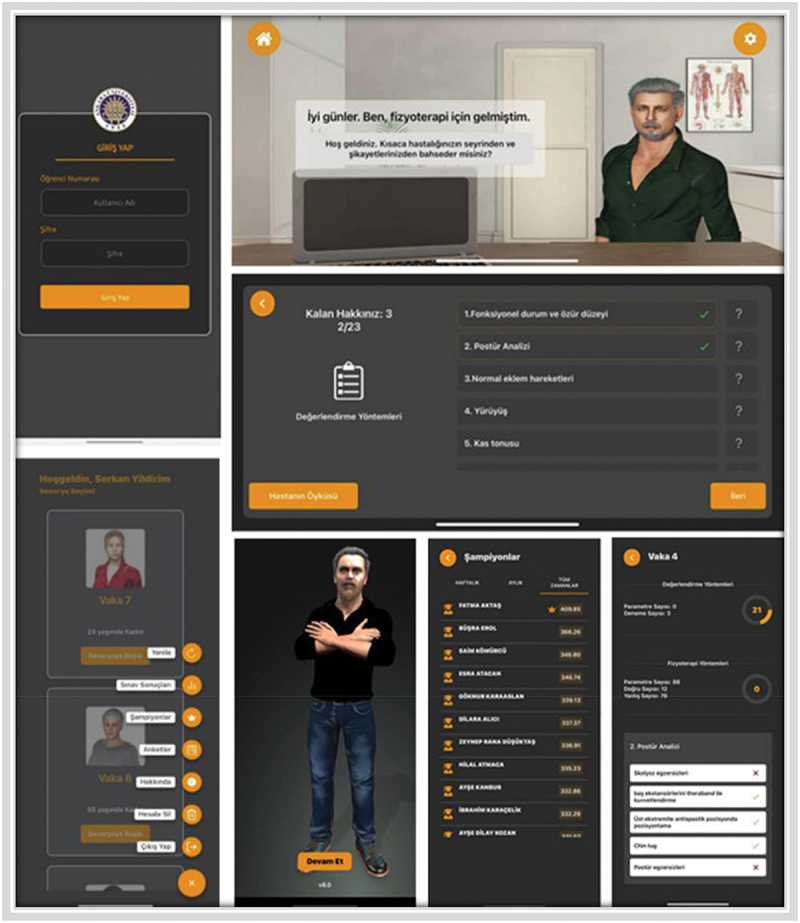
Images from the application.

The suitability of the case and application contents, the visuals of which are presented in Figure 1, was determined by obtaining expert opinions through the coding keys created. Before the experimental process related to the mobile application, which was finalized with the consensus of all experts, the experimental group was informed about the aim of the study and asked to download the application on their smartphones and sign up to become a member. The guidelines for the use of the mobile application and instructions for the management of cases were also included in the process. After this last session of sharing information, the process was not interrupted in any way.

### Data collection

The quantitative data were collected through an achievement test with five multiple-choice questions given before and after the intervention. Following the analysis of the data obtained after the first administration of the achievement test consisting of 32 questions and the consensus of the experts at the pretest (17 questions) stage, the experimental group was given information about the mobile application. After the information session, the participants were asked to become a member by downloading the mobile application to their smartphones, and the intervention stage of the study began. The pretest and posttest were conducted 91 days apart, and the study was terminated after the posttest (32 questions), which included the questions asked in the pretest. The qualitative data were collected from 26 students in the experimental group through an open-ended questionnaire. In this last stage, the students were instructed to respond to the following instruction: ‘Write your opinions and suggestions, if any, about the mobile virtual patient application.’ This instruction, which was provided to reveal their opinions about the intervention, was given in the classroom and under the researcher’s supervision, to be answered in writing, to allow students express themselves freely and in detail.

### Data analysis

The achievement test, which included multiple-choice questions, was administered at the same time to the experimental and control groups at the beginning and end of the study period. In the achievement test, 1 point was given for each correct question and 0 point was given for each incorrect or blank question. Since there was homogeneity between the groups, the distribution met the normality assumption, and there were two groups, parametric tests were conducted to analyze the data. In assessing the efficacy of the respective environments, a 2 × 2 split plot design was employed, while a two-factor analysis of variance incorporating mixed measures was implemented to interrogate the primary research query. The accuracy of the results of the analysis of the effect of the mobile application as an assistive technology in addition to the traditional environment on students’ achievement was analyzed in a different quantitative analysis with a second dataset. Thus, the reliability of the study was increased by verifying the same research question with a different quantitative analysis technique. To do this, the achievement test consisting of a total of 32 questions, the reliability of which was tested with all the questions included in the pretest stage, was given as a posttest and the data were analyzed by t-test analysis in independent measurements. In the study, Cohen’s d effect size analysis was performed to determine the extent to which the mobile virtual patient application in addition to the traditional environment affected the achievement. Based on benchmarks proposed by Cohen [43], effect sizes are often classified as small (d = 0.2), medium (d = 0.5), or large (d = 0.8).

In the categorical analysis process of the qualitative part of our study, in which we administered an open-ended question form to the experimental group, we meticulously followed several key steps, as outlined by Corbin & Strauss [44]. The data were manually coded, enabling us to identify emerging themes. This process involved creating categories based on these themes, and then organizing and refining these categories to best represent the data. Each category was thoroughly reviewed to ensure it accurately captured the essence of the corresponding data. The findings were then defined and interpreted, with each researcher bringing their own perspective to the analysis. This manual approach allowed for a nuanced and in-depth exploration of the qualitative data. To ensure data trustworthiness and consistency in our analysis, each researcher independently analyzed the data. Discrepancies were resolved through discussion and consensus among the research team, enhancing the reliability of our findings. No specific software was used for the qualitative analysis; instead, we relied on manual methods. Additionally, frequency analysis, as described by Ryan & Bernard [45], was used to quantitatively determine the intensity and importance of particular items in the data.

## Results

To establish a baseline for comparing the effectiveness of different learning environments, a pretest was administered to the students before the study. This pretest aimed to determine the prior knowledge of the two groups and to test the homogeneity and normality of the distribution. The pretest results indicated homogeneity between the groups (Levene’s Test F = 1.351, *p* > .05). Normality tests revealed that the pretest measurement values for the control group (Skewness=.329, Kurtosis=−.689) and the experimental group (Skewness=.947, Kurtosis = .705) were normally distributed. An independent samples t-test was performed to identify any significant differences in the pretest scores of both groups before the experimental procedure, which found that the difference between the arithmetic mean scores was not significant (t(50)=-.051, *p* = .959). Based on these results, the students were categorized into two groups: experimental and control.

A 2 × 2 split plot design assessed how effective different learning environments were for students. In this design, the first factor refers to two different experimental environments (traditional environment – environment in which mobile virtual patient application is used as an additional assistive technology to the traditional environment) and the second factor refers to pre- and post-experimental measurements (pretest-posttest). Two-factor analysis of variance for mixed measures was used to analyze this research question. The pretest-posttest mean scores and standard deviation values of the environments in which the students studied are given in Table 3.

**Table 3.** Learning environments mean and Standard Deviation Values (AT-17).

Group	N	Pretest(17)		Posttest (17)		Effect size	
		$\overset{ˉ}{X}$	S	$\overset{ˉ}{X}$	S	Cohens’d	Level
Control	26	5.58	2.08	6.58	2.23	.464	Small
Experimental	26	5.54	3.23	9.46	3.22	1.215	Large

According to the results obtained, while the average achievement score of the students taught in the traditional environment was 5.58 prior to the experiment, this value was 6.58 subsequent to the experiment. The average achievement scores of the students in the environment where the mobile application was used as an assistive technology besides the traditional environment were 5.54 and 9.46, respectively. As such, an increase is observed in the achievement scores of the students studying in both the traditional environment and the mobile application environment. The two-factor ANOVA results of whether the changes observed in the achievement of the students in two different environments show a significant difference are presented in Table 4.

**Table 4.** ANOVA results regarding learning environments.

Source of variance	Sum of squares	Sd	Mean squares	F	p	eta
Between subjects	471.346	51				
Group (Individual/Group)	52.654	1	52.654	28.600	.015	.112
Error	418.692	50	8.374			
Within subjects	545.999	52				
Measurement (Pretest-Posttest)	157.538	1	157.538	23.660	<.001	.321
Group*Measurement	55.538	1	55.538	8.340	.006*	.143
Error	332.923	50	6.658			
Total	1071.345	103				

*p < .05.

According to the results obtained, from pre-experiment to post-experiment, the success of the students differed significantly, in other words, the combined effects of being in different learning environments and repeated measures factors on achievement were significant (F(1–50) = 8.34, *p* = .006). This observation suggests that the distinct learning environments exert differential impacts on student achievement. It was found that the environment in which the achievement scores increased more than before the experiment, in which the mobile application was used as an additional assistive technology to the traditional environment, was more effective in increasing the achievement of the students. When the effect of the environment on the change in their achievements was examined, it was seen that while the effect size of the students in the control group was small (d = .464), it was determined to be large (d = 1.215) for those in the experimental group. To increase the accuracy and reliability of the result indicating that the environment in which the mobile application is used as an additional assistive technology to the traditional environment is more effective in increasing student success, t-test analysis was performed. The results of the analysis obtained from the achievement test data consisting of a total of 32 questions whose reliability was tested, and 17 questions used in the pretest stage of the study were included without any changes, as given in Table 5.

**Table 5.** T-test results for the posttest achievement scores of the students in the control and experimental groups (AT-32).

Group	N	$\overset{ˉ}{X}$	S	Sd	t	p	Cohen’d
Control	26	17.00	3.78	50	4.091	<.001*	1.135
Experimental	26	22.38	5.54				

*p < .05.

The analysis of the posttest data showed a significant difference between the achievement scores of the experimental and control groups (t_(50)_ = 4.09, *p* < .001). While the average achievement score of the students of the control group students was 17.00, the experimental group using the mobile virtual patient application in addition to the traditional learning process was 22.38. The use of mobile virtual applications in addition to the traditional environment were found to have a large (d = 1.135) effect on the clinical reasoning success of students. These results show that the use of mobile virtual patient application in addition to the traditional learning process is effective in increasing achievement, and different quantitative data analysis results support each other.

The opinions of the experimental group about the mobile virtual patient application and the intervention process were collected through an open-ended question form. The written data obtained were analyzed by categorical analysis and frequency analysis techniques, which are types of content analysis, and the findings are presented in Table 6.

**Table 6.** Students’ mobile virtual patient application and their views on the process*.

Positive	*n*	*%*
The stories and cases presented were realistic.	25	96.2
The story presented made it easy for me to learn about the organization.	24	92.3
It contributed to the integration of the theoretical knowledge we gained in the course with practice.	23	88.5
I think it will increase our clinical competence.	21	80.8
I felt progress in my case-solving ability.	19	73.1
It helped me increase my self-confidence before meeting a real patient.	17	65.4
Negative		
I had a hard time working on the small screen.	3	11.5

*Multiple answers are possible.

The qualitative data analysis revealed that the students’ opinions about the mobile virtual patient application were quite positive. When the opinions of the students taken with the open-ended question form were analyzed, it was determined that they generally included more than one positive factor. Their views indicating that the stories and cases presented through the application create a perception of reality underscore its contribution to the learning of the story organization. S12’s statement on the subject, ‘ … *the system is realistic … it provided the opportunity to practice what I saw in the course. This makes us feel like we did some internships*.’ Thus, it was determined that the students gave positive feedback that it contributed to the integration of theoretical knowledge with practice. Furthermore, it was frequently stated by the students that the practice contributed positively to the case analysis as well as to gaining clinical competence. In addition to these, S19 made the following statement: ‘ … *I have gained pre-clinical case solving ability. … meanwhile, I felt like I met a real patient … if I study more cases, I will feel better with real patients.’* As such, they expressed a positive opinion that it was effective in increasing their self-confidence, especially regarding meeting with the real patient. However, S07 stated the following: *‘ … it made me practice. … it was realistic, but because my phone’s screen is small, I had difficulty in transitions’*, and it was observed that there were also a few students who had problems with the screen size.

## Discussion, conclusion and suggestions

### Discussion and conclusion

A pretest-posttest control group quasi-experimental design was included to determine the effect of using the mobile virtual patient application, which includes narrated case-based virtual patients, as an assistive technology on the change in students’ clinical reasoning skills. The results of the academic achievement test developed by the researchers to assess changes in students’ clinical reasoning skills indicated that students who experienced both traditional teaching methods and the mobile virtual patient application demonstrated a significant increase in their achievement scores. Based on the analyses made on whether the changes in question showed a significant difference, it was determined that the success of the students studying in the two environments differed significantly from the pre-experiment to the post-experiment stage. This finding shows that each of the learning environments has different effects on increasing students’ achievement, and supporting the traditional environment with mobile virtual patient application is more effective in increasing achievement. The analysis of the posttest data, which included the questions asked in the pretest stage of the study, as well as additional questions that the students encountered for the first time, revealed a significant difference between the achievement scores of the experimental and control groups. These findings underscore the effectiveness of integrating mobile virtual patient applications into traditional learning environments to enhance students’ clinical reasoning skills. The results also indicate that the practice has the potential to increase result-oriented academic achievement, which includes clinical reasoning skills. Considering that clinical reasoning is a context-dependent skill, evaluating case-specific outcomes might be more appropriate for its measurement [6]. While suggesting the potential of testing the mobile virtual patient application, our study also tested the impact of these skills on outcome-focused academic achievement through questions encompassing clinical reasoning skills. According to the findings of Torres et al. [18], methodologies that incorporate interactive virtual scenarios have been demonstrated to enhance clinical reasoning capabilities. Research in health professions education suggests that virtual tools for patient education are effective for enhancing clinical reasoning, especially when they offer feedback and opportunities for reflection [25,27,46]. Furthermore, the results of our study align with existing literature in the field of health professions education, reinforcing the growing body of evidence that supports the effectiveness of digital tools in enhancing clinical reasoning skills. Similar improvements in clinical reasoning and other cognitive skills among students using interactive and technology-assisted learning methods have been reported (e.g [25,47–49]). This consistency with prior research not only validates our findings but also contributes to the broader understanding of how innovative educational technologies can be effectively integrated into healthcare education.

The mobile virtual patient application and the opinions about the process collected from the students through an open-ended question form generally support the increase in academic achievement related to clinical reasoning skills. The results obtained at this stage of the study, in which the analysis was carried out with content analysis, show that almost all of the students exhibited a positive attitude towards the mobile virtual patient application. The students, who mentioned more than one positive factor related to the practice, stressed its contribution to the learning of the story organization, along with their opinions that the presented stories and cases create a perception of reality. These findings provide additional evidence of the effectiveness of case-based learning in enhancing clinical reasoning skills, corroborated by studies [47,50,51]. According to the findings, the use of narrated case-based scenarios in the mobile virtual patient application actively engaged students in clinical reasoning and critical thinking before encountering real patients, aligning with Cook and Triola’s (2019) observations. Students’ feedback regarding the application’s contribution to integrating theoretical knowledge with practice was positive. They frequently stated that the application contributed significantly to both case analysis and the development of clinical competence. In addition to these, most of the students expressed positive opinions that it was effective in increasing their self-confidence in meeting with the real patient. All these results indicate that the application supports clinical judgment, provides practical experience, improves academic achievement, and contributes positively to motivation. Considering the effectiveness of technology-mediated learning, student satisfaction has been identified as a critical factor [18,52], and student satisfaction is generally reflected in the academic success of clinical reasoning skills. Moreover, studies have shown that virtual patients enhance active learning, thereby fostering the development and assessment of clinical reasoning skills [25,47,53]. Based on all these results, it can be argued that in today’s world where mobile learning has become a new trend in health education, mobile learning can be an assistive technology to eliminate integration problems and to equip future health professionals with high-level knowledge and skills on subjects such as learning new medical procedures and treatment planning.

Despite these promising findings, it is important to acknowledge the limitations of this study. The sample size and its composition may limit the generalizability of the findings. The quasi-experimental design, though robust, does not offer the same level of control as randomized controlled trials. Current and future technological variations in the use of the mobile virtual patient application could also impact the outcomes. Furthermore, the use of self-reported measures may introduce some biases, although efforts were made to minimize their impact. Additionally, the measurement instrument used in this study, while carefully developed and tested, may have inherent limitations in terms of validity or reliability that could have affected the findings. The findings are specific to the context of this study and may not be directly applicable to other educational settings.

## Suggestions

In light of the observed findings, the subsequent recommendations are proposed:

- Narrated case-based mobile virtual patient applications can be used to improve students’ clinical reasoning skills.
- Case-specific results may also be more appropriate to measure clinical judgment, as clinical judgment is a context-dependent skill.
- Educators’ opinions should be sought on the use of the developed mobile virtual patient application in learning environments and their involvement in the process should be ensured.
- It should be considered as a prerequisite for successful practices that students have mobile devices that they can use to run the mobile virtual patient application developed without any problems, and that adequate IT infrastructure and equipment (internet-connection speed, etc.) are provided.
- It would be appropriate to test the study/practice in other courses that require or aim to develop clinical reasoning skills.

## Data Availability

The datasets generated during and/or analysed during the current study are available from the corresponding author on reasonable request.

## Data availability statement

All data generated and analyzed during this study are included in this published article.
